# Estimating medication adherence from Electronic Health Records: comparing methods for mining and processing asthma treatment prescriptions

**DOI:** 10.1186/s12874-023-01935-3

**Published:** 2023-07-12

**Authors:** Holly Tibble, Aziz Sheikh, Athanasios Tsanas

**Affiliations:** 1 Usher Institute, University of Edinburgh, Edinburgh, Scotland; 2 Asthma UK Centre for Applied Research, University of Edinburgh, Edinburgh, Scotland

**Keywords:** Adherence, Asthma, Electronic Health Records, Compliance, Corticosteroid

## Abstract

**Background:**

Medication adherence is usually defined as the extent of the agreement between the medication regimen agreed to by patients with their healthcare provider and the real-world implementation. Proactive identification of those with poor adherence may be useful to identify those with poor disease control and offers the opportunity for ameliorative action. Adherence can be estimated from Electronic Health Records (EHRs) by comparing medication dispensing records to the prescribed regimen. Several methods have been developed in the literature to infer adherence from EHRs, however there is no clear consensus on what should be considered the gold standard in each use case.

Our objectives were to critically evaluate different measures of medication adherence in a large longitudinal Scottish EHR dataset. We used asthma, a chronic condition with high prevalence and high rates of non-adherence, as a case study.

**Methods:**

Over 1.6 million asthma controllers were prescribed for our cohort of 91,334 individuals, between January 2009 and March 2017. Eight adherence measures were calculated, and different approaches to estimating the amount of medication supply available at any time were compared.

**Results:**

Estimates from different measures of adherence varied substantially. Three of the main drivers of the differences between adherence measures were the expected duration (if taken as in accordance with the dose directions), whether there was overlapping supply between prescriptions, and whether treatment had been discontinued. However, there are also wider, study-related, factors which are crucial to consider when comparing the adherence measures.

**Conclusions:**

We evaluated the limitations of various medication adherence measures, and highlight key considerations about the underlying data, condition, and population to guide researchers choose appropriate adherence measures. This guidance will enable researchers to make more informed decisions about the methodology they employ, ensuring that adherence is captured in the most meaningful way for their particular application needs.

**Supplementary Information:**

The online version contains supplementary material available at 10.1186/s12874-023-01935-3.

## Background

Medication adherence is defined as the alignment between how a patient takes their medication, and the regimen agreed to with their healthcare provider [1]. Non-adherence to treatment is a substantial impediment to treatment effectiveness [2–7], and subsequent poor clinical outcomes may lead to unnecessary dose escalation and/or additional treatment to control symptoms, which could result in avoidable side-effects [8–12]. Estimates of non-adherence incidence are crucial for identifying the most at-risk patients [13–15], approximating associated costs (both financial and quality of life) [16–20], and accurately assessing the effectiveness of new treatments [21–24]. Electronic Health Records (EHRs) provide the opportunity to estimate adherence cost-effectively and at scale, although practically not all aspects of adherence can be measured (such as whether the medication is being taken once collected) [25].

“One challenge in studying varying [adherence] is that no single feature can express it.” Boissel et al. [26].

Vollmer et al*.* [27] defined eight Continuous, Multiple Interval, Measures of Medication Availability (CMAs), calculated using multiple refills, labelled CMA1 to CMA8 (summarised in Table 1). CMA measures 1 through 4 are explicitly measures of medication acquisition rather than medication taking, as they use the amount of medication obtained over a period, rather than any calculations requiring acknowledgement of the spacing and gaps in availability. This makes them relatively simple to calculate but results in an overly simplified summary of the observed time series. In contrast, CMA5 to CMA8 incorporate the timing of the prescriptions (all at once, or evenly spaced) within the observation period to better detect gaps in medication availability. This inhibits them from detecting over-supply of medications, marking that a patient is using their medication at more regular intervals or dosages than they had been instructed [25].

**Table 1:** Start and end of analysis window within observation period for continuous, multiple interval, measures of medication availability

Adherence Measure	Start of Window	End of Window	Derivation
CMA1	Day of first prescription	Day before final prescription	$\frac{\mathrm{Supply days obtained in window}}{\mathrm{Window duration}}$
CMA2	Day of first prescription	End of observation period	$\frac{\mathrm{Supply days obtained in window}}{\mathrm{Window duration}}$
CMA3	Day of first prescription	Day before final prescription	﻿minimum (CMA1, 1)
CMA4	Day of first prescription	End of observation period	﻿minimum (CMA2, 1)
CMA5	Day of first prescription	Day before final prescription	$\frac{\mathrm{Days with medication available in window}}{\mathrm{Window duration}}$
CMA6	Day of first prescription	End of observation period	$\frac{\mathrm{Days with medication available in window}}{\mathrm{Window duration}}$
CMA7	Start of observation period	End of observation period	$\frac{{\mathrm{Days with medication available }}^{a}\mathrm{ in window}}{\mathrm{Window duration}}$
CMA8	Day that supply that was available at the start of observation period theoretically exhausted	End of observation period	$\frac{{\mathrm{Days with medication available }}^{b}\mathrm{ in window}}{\mathrm{Window duration}}$

^a^accounting for supply remaining, at the start of observation from dispensings prior to observation

^b^accounting for supply obtained between the start of observation and the start of the window

There is currently no pragmatic guidance on the most appropriate method for measuring adherence in specific cases [28–30]. There are very few studies that have reported more than one adherence measure [3], which makes direct comparisons challenging. Additionally, it is becoming increasingly common to encourage asthma patients to self-manage their treatment to some extent, and use their controller inhaler only *as needed* [31–33]. Such patients can be flagged using dosage instructions recorded in prescription records. For those patients, these measures may be considered a proxy for medical usage patterns, rather than as a measure towards ‘optimal’ adherence [34], but they may still have some predictive value for future outcomes including asthma attacks.

The aim of this study was to demonstrate how different adherence measures present in real world scenarios to facilitate the selection of the most appropriate adherence measure for a given study. We used asthma, a chronic and highly prevalent respiratory disorder [35, 36] with typically high rates of non-adherence [37–42], as a case study to highlight some of the disease specific aspects that researchers must consider. Asthma can be effectively managed in the majority of individuals through regular use of Inhaled CorticoSteroids (ICS) [43–45], although additional therapies may be used in parallel in those with insufficient control of their symptoms. ICS medications are also available in combination (within a single inhaler) with Long-Acting Beta-2 Agonist (LABA) [46].

Our objectives were to (1) critically evaluate different adherence measures of medication adherence in a Scottish EHR dataset, and (2) guide the selection of the most appropriate adherence measure towards specific aims, such as detecting change in patient behaviour and predicting clinical outcomes.

## Methods

### Data

The Asthma Learning Healthcare System (ALHS) dataset was created to develop and validate a prototype *learning healthcare system* for asthma patients in Scotland, in which patient data are used to generate a continuous loop of knowledge-generation, evidence based clinical practice change, and change assessment/validation [47]. Over half a million patients from 75 general practices in Scotland were recruited, with primary care records linked to national accident and emergency, hospital and mortality datasets using the Scottish health identification number known as the Community Health Index (CHI).

Prescription dispensing data were available in the Scottish Prescribing Information System (PIS) dataset, which is described elsewhere [48]. In Scotland, prescription medications (which are free, unlike in many other countries including within the UK) are requested and approved in primary care, and then the patient requests the prescription be dispensed at a pharmacy of their choice. Unlike in other parts of the UK [49], prescribing and dispensing records can be routinely linked in Scotland, as each prescription has a unique identifier which is carrier forward to the dispensing record. Primary care prescribing records from January 2009 to March 2017 were linked to dispensing data so that only collected prescriptions were included. The fields available in the prescription dataset were: pseudo-anonymised patient study identifier (such as “ID0001” – allowing linkage between datasets and for observations within datasets, without revealing the identity of the individual), date of prescription, date of dispensing, medication name, BNF item code, formulation, prescribed quantity, dispensed quantity, and free-text native dose instructions.

### Prescription supply handling approaches

To estimate adherence, we first needed to calculate when a medication supply would be exhausted provided it is used according to the dose directions. The expected supply duration of a prescription is a function of the amount that should be taken every day (the frequency of doses per day and the dose quantity) and the volume of the prescription (see Additional file 1: Appendix A). For example, a 60-dose inhaler prescribed to be taken with two puffs twice a day will last for 15 days if taken as directed.

The definition of each CMA provided by Vollmer et al*.* [27] states that in CMA5 to CMA8 the number of days of *theoretical use* should ﻿assume “*medications taken as directed and new medications banked until needed*”. We have included two variations of this approach to estimating medication supply, and compared the findings to when previous supply were discarded when a new prescription was obtained:

Discard: Assuming all medication was lost or disposed of at a new prescription (ignoring leftovers) and calculated using only the time since the last dispensing, and how much was dispensed.

Cap: Assuming the maximum amount available after a dispensing was double the amount dispensed (capping the leftovers)

Retain: Assuming all leftovers were available, and no medication was ever lost or disposed of (as used by Vollmer et al. [27])

The supply estimation method will henceforth be denoted by a subscript of D (discard), C (cap) or R (retain). For example, CMA5_D_ denotes CMA5 with over-supply discarded.

Multiple prescriptions obtained on the same day were condensed into a single record by summing the supply obtained and removing the first record (which would have the refill interval duration calculated as zero days). Changes in medication (therapy type, strength, brand, etc.) were disregarded in this analysis, for all CMAs, thus remaining supply of medicine was not automatically assumed to be binned if the primary medicine was changed. Instead, any medication remaining in supply at the event of a new and changed prescription being dispensed was handled in the same three ways as any other remaining supply – either discarded, capped, or retained. Changes to the number of doses to be taken each day, however, were assumed to come into effect immediately, even in cases when the carryover supply was for a different medication as well as daily dosing regimen.

### Analysis plan

The estimates of each adherence measure, for each supply handling approach, were estimated over three intervals: the patient’s full follow-up, by year, and by year-quarter. CMA7 and CMA8 could only be calculated when some prior history of medication was known, such that the supply quantity at the start of the observation period can be calculated. For this analysis, these two measures were not calculated in the first year or quarter of follow-up (no carry over was assumed for the full follow-up estimate).

For each adherence measure, we computed the Spearman correlation coefficients between one interval (year or quarter) and the next. Correlation coefficients were considered to denote statistically strong (|R|> 0.7), moderate (0.3 <|R|< 0.7), or weak (|R|< 0.3) statistical associations.

## Results

### Prescription records by analysis time-intervals

After initial cleaning, 1,627,626 ICS or ICS + LABA prescription records were identified for 91,334 unique individuals. 15,224 people (16.7%) had only a single prescription during their observation period; their CMA1, CMA3, and CMA5s could not be calculated for any interval length. 52 people had only a single prescription, which was recorded on the final day of their observation period, resulting in a total follow-up time of zero days. The median follow-up was 7.1 years, with an interquartile range of 4.2 to 8.0 years (maximum 8.2 years).

There were 382,105 person-years containing at least one prescription, of which 92,948 (24.3%) contained only one (and thus CMA1, CMA3, and the CMA5s could not be calculated). The median number of prescriptions filled in a year (containing at least one prescription) was 3, with a range of 1 to 67 (interquartile range 2 – 6). Similarly, there were 1,002,729 person-quarters in which at least one prescription was filled, of which 579,664 (57.8%) contained only one prescription fill. The median number of prescriptions filled in a quarter-year (containing at least one prescription) was 1, with a range of 1 to 19 (interquartile range 1 – 2).

### CMA adherence measures

CMA1 and CMA2, which are not constrained to the [0,1] range like the other adherence measures, are presented in Table 2. We can see that as the interval of the time window decreases in length from the full follow-up to by years and by quarter-years, the median measure value of both measures increased. For quarter-years in which there were multiple prescriptions (and a value could thus be calculated), the lower interquartile was above 1 for both measures, indicating that more medication was being collected than was required for that period.

**Table 2:** Median and spread of CMA1 and CMA2 across time windows

Measure	Time Window	Median	Interquartile Range	Range
CMA1	All of follow-up	0.69	0.38 – 1.07	0.01—120
	Years	1.20	0.85 – 1.74	0.08—550
	Quarter-Years	2.40	1.67 – 3.70	0.23—550
CMA2	All of follow-up	0.36	0.12 – 0.76	< 0.01 – 150
	Years	0.86	0.50 – 1.28	0.03 – 360
	Quarter-Years	1.62	1.08 – 2.76	0.07 – 720

CMA1 and CMA2 have values in the range [0,∞), in which a value of 1 represents ‘perfect’ adherence and values over 1 represent ‘excessive’ adherence, or medication oversupply

**Fig. 1:**
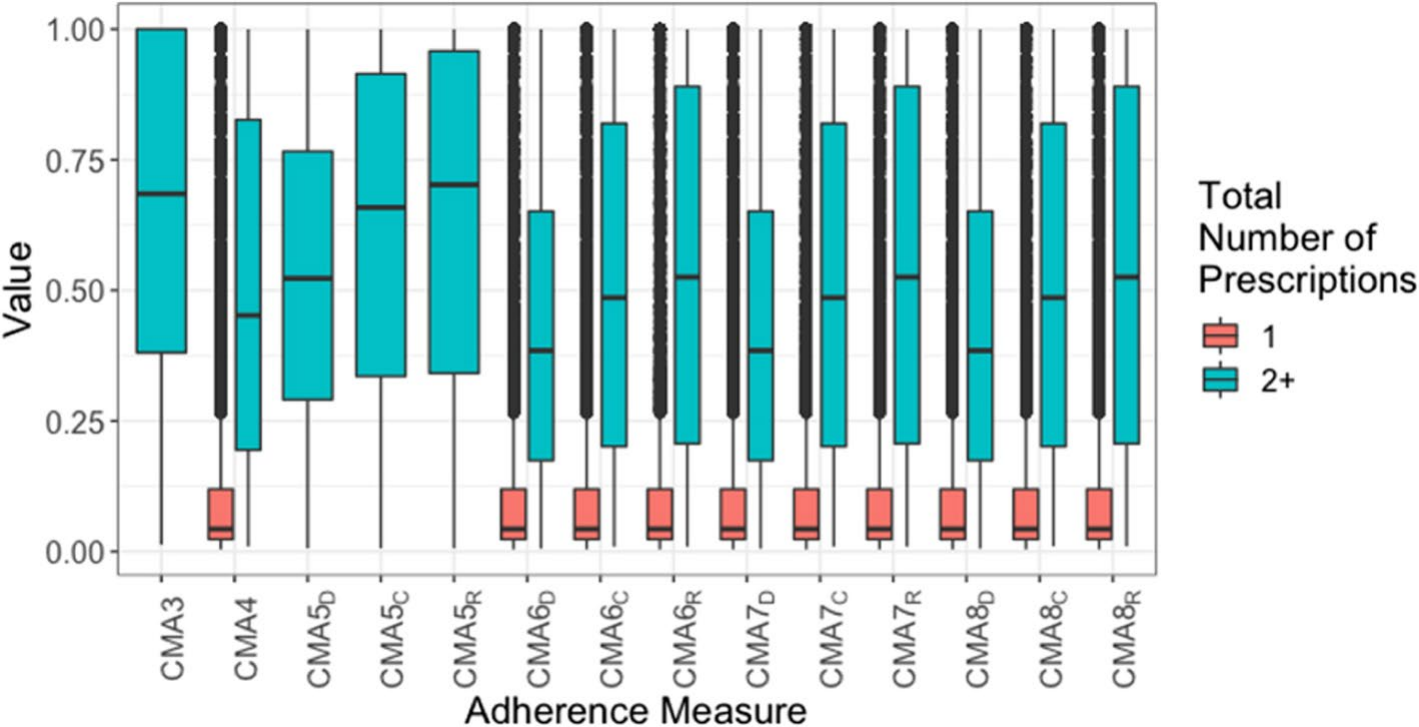
Boxplots of bounded CMAs for the full follow-up, for those with single or multiple (2 or more) prescriptions. Note: CMAs 1–4 are measures of medication acquisition while CMAs 5–8 are measures of medication availability. For the latter, the subscript denotes the oversupply handling approach: Discarding all remaining medication, Retaining all remaining medication, Capping the amount of remaining medication

**Fig. 2:**
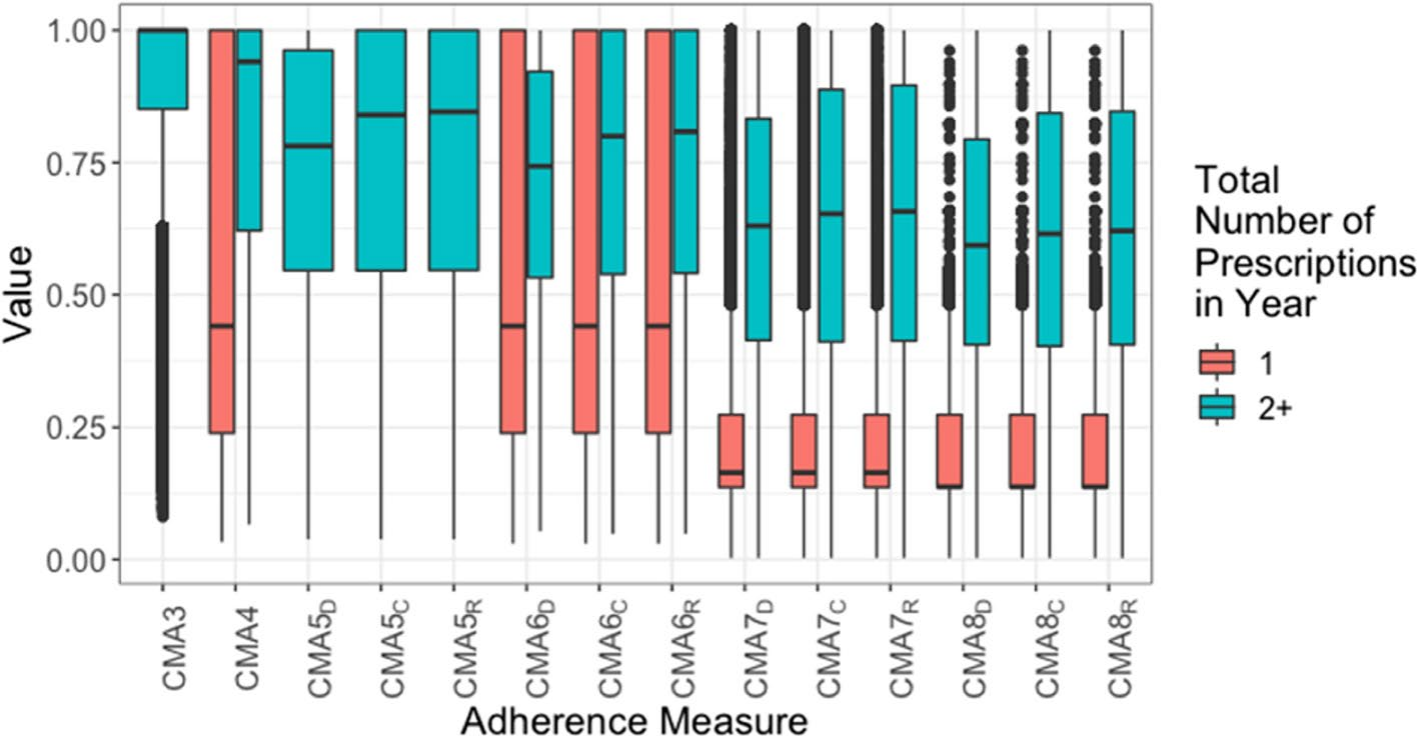
Boxplots of bounded CMAs for each person-year, for those with single or multiple (2 or more) prescriptions. Note: CMAs 1–4 are measures of medication acquisition while CMAs 5–8 are measures of medication availability. For the latter, the subscript denotes the oversupply handling approach: Discarding all remaining medication, Retaining all remaining medication, Capping the amount of remaining medication

**Fig. 3:**
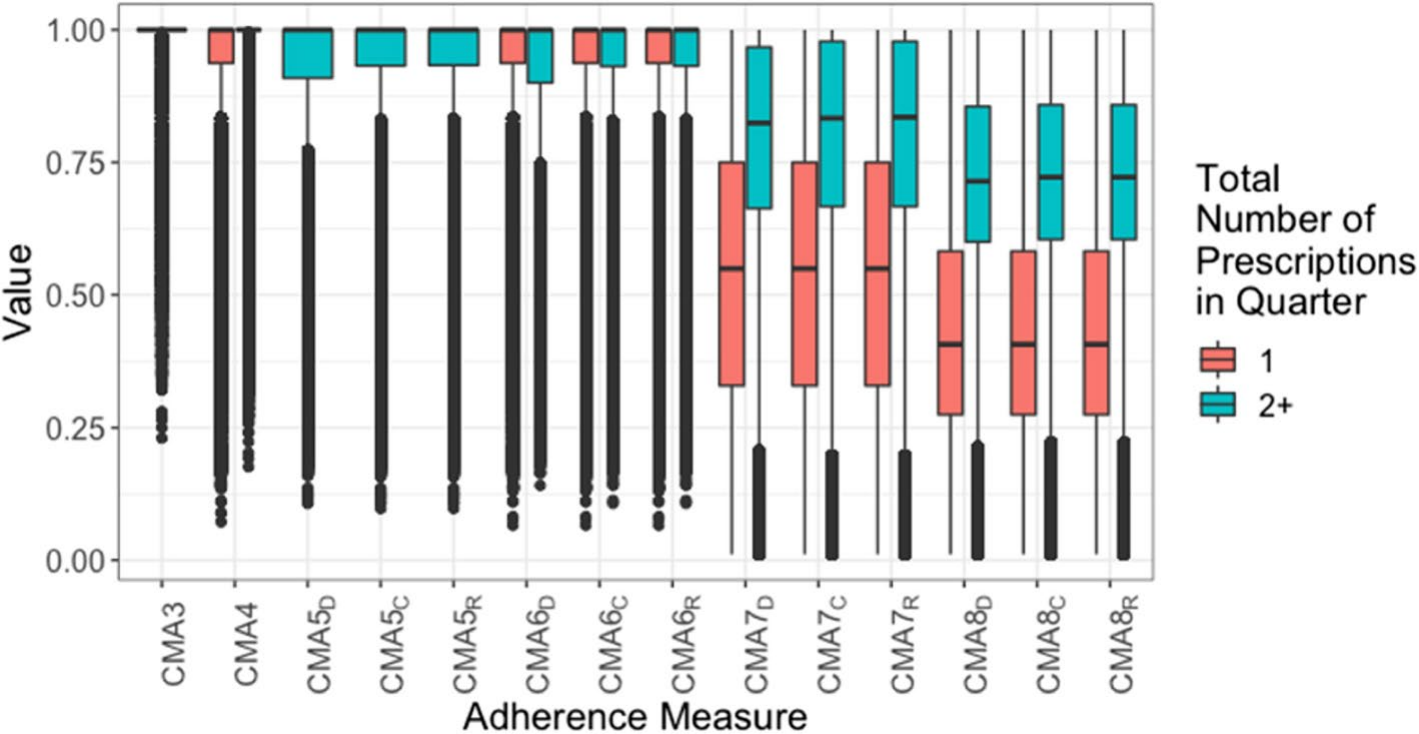
Boxplots of bounded CMAs for each person-quarter, for those with single or multiple (2 or more) prescriptions. Note: CMAs 1–4 are measures of medication acquisition while CMAs 5–8 are measures of medication availability. For the latter, the subscript denotes the oversupply handling approach: Discarding all remaining medication, Retaining all remaining medication, Capping the amount of remaining medication

Figures 1, 2 and 3 show boxplots of CMAs 3 to 8, for each time window, respectively. Like CMA1, CMAs 3 and 5 can only be calculated if there were at least two prescriptions in each time window (such that there is at least one prescription interval with a known end date). As such, estimates for those with only one prescription in the interval have been plotted separately (in coral) to the estimates for those with two or more prescriptions in the time period (in teal), so that distributions for the same population are being compared.

Across the full follow-up (Fig. 1), the average for each adherence measure was higher when over-supply was allowed (subscript R). This was also true at the year-level (Fig. 2), however there was less distinction, and the capped over-supply (subscript C) closely resembled the uncapped over-supply in distribution. In the cohort with multiple prescriptions each year, especially high (outlier) quantities of over-supply were uncommon. At the quarter-year level (Fig. 3), there was no distinction between the estimates according to supply-handling method.

At both the full follow-up and year-level, all adherence measures have a similarly wide range. For CMA2, CMA5s and CMA6s, most person-quarters have over 85% estimated adherence, and many values lower than that are classed as outliers. For CMA7s and CMA8s, however, there is a much wider spread of values, meaning that these measures may provide a more nuanced interpretation of the adherence during this period.

### Correlation of CMA measures between subsequent time intervals

Table 3 lists the Spearman correlation coefficient for pairs of adherence measure estimates in individuals with estimates in two (or three) chronologically adjacent time windows (years or quarters). For example, there were 154,189 person-years for which CMA1 could be estimated in both that year, and the following two years. The Spearman correlation coefficient between the first and third years was 0.540.

**Table 3:**
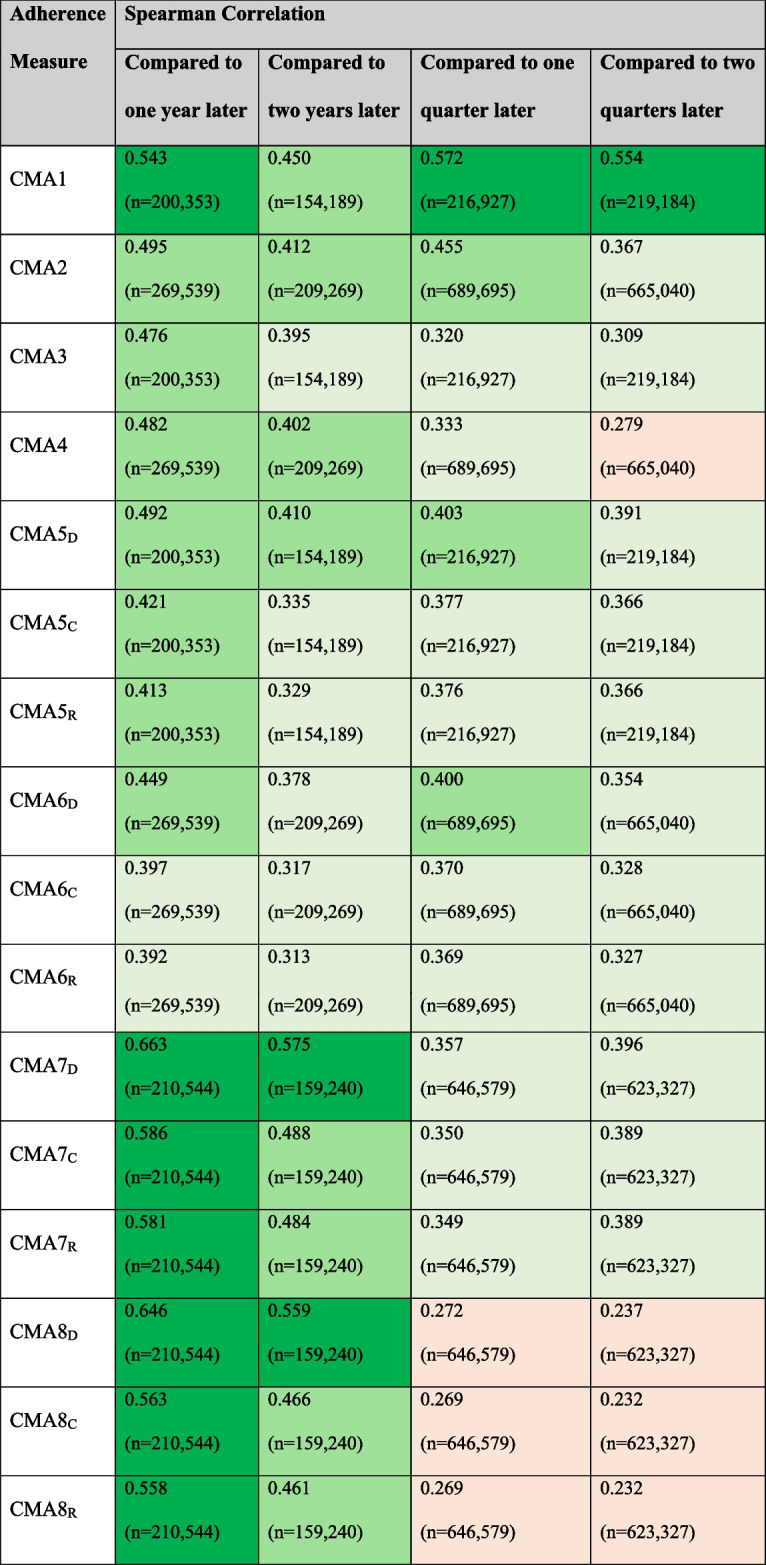
Spearman correlation between multiple prescription adherence measures for subsequent intervals (years and quarters)

CMAs 1–4 are measures of medication acquisition while CMAs 5–8 are measures of medication availability. For the latter, the subscript denotes the oversupply handling approach: Discarding all remaining medication, Retaining all remaining medication, Capping the amount of remaining medication. CMA1 and CMA2 have values in the range [0,∞), While CMAs 3 to 8 have values in the range [0,1]. For all measures, a value of 1 represents ‘perfect’ adherence. Values over 1 for CMA1 and CMA2 represent ‘excessive’ adherence, or medication oversupply. All correlation coefficients were statistically significant (*p* < 0.05). Colour coding key for magnitude of correlation coefficient: light orange – less than 0.3, light green – 0.3 to 0.4, medium green – 0.4 to 0.5, dark green – over 0.5

The Spearman correlation between the estimate in one year and the next (when both were estimable for an individual) was highest for CMA1, the CMA7s, and CMA8_D_ (Table 3). In the CMAs 5 to 8, the estimates when leftovers were discarded (subscript D) were consistently the most correlated with the next year’s estimate. When estimates were compared to the estimate two years in the future, the CMA7_D_ and CMA8_D_ were still the most strongly correlated.

Comparing subsequent quarter-years, we can see that CMA1 is still the most highly correlated, but that CMAs 7 and 8 are substantially lower. CMA3 has also substantially lower correlation than CMA1, as CMA3 is simply CMA1 capped at 1. This highlights again how commonly CMA1 is identifying oversupply at the quarterly level, as it requires multiple prescriptions within a 3-month period, as discussed around Table 2.

### Correlation of CMA measures within time intervals

**Fig. 4:**
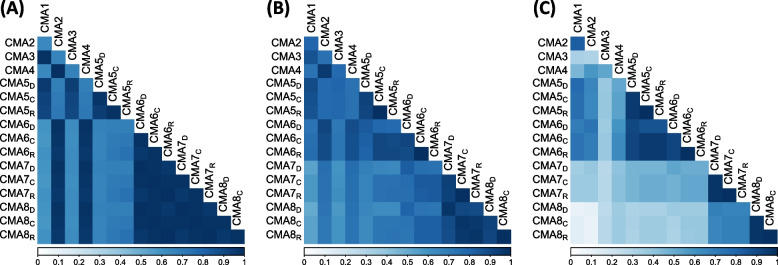
Correlation Matrix of adherence measures matched by period: (**A**) all of follow-up, (**B**) calendar year, (**C**) calendar quarter-year. Note: CMAs 1–4 are measures of medication acquisition while CMAs 5–8 are measures of medication availability. For the latter, the subscript denotes the oversupply handling approach: Discarding all remaining medication, Retaining all remaining medication, Capping the amount of remaining medication

Across an individual’s full follow-up, there was moderate (0.3 <|R|< 0.7) or strong (|R|> 0.7) positive correlation between all adherence estimates (Fig. 4A, minimum Spearman correlation coefficient = 0.59). All pairwise combinations within the same requirement class (e.g., CMA1, CMA3, and CMA5s require there to be at least two prescriptions) had correlation greater than 0.9, and all combinations between measures of different requirement classes had correlation coefficients under 0.75. The coefficient was only calculable for each non-missing pair of records, and as such only includes those with at least 2 prescriptions during follow-up.

In Fig. 4B, which shows the correlation coefficients between pairs of measures at the yearly level, we can see that this relationship between requirement class and correlation coefficients has become less pivotal. All pairwise combinations were still considered to have either moderate or strong positive correlation (minimum correlation coefficient = 0.53). For the CMA5 to 8 s, the correlation coefficients between supply estimation approaches CAP and RETAIN are consistently higher than between approaches DISCARD and CAP or DISCARD and RETAIN.

In quarterly estimate comparisons, in Fig. 4C, the differences between the measures are far more substantial. The minimum correlation coefficient was now 0.14, and 10% of the comparisons (*n *= 12/120) found only weak positive correlation (0 <|R|< 0.3). Despite this, we still observed strong *internal* correlation (between supply estimates) for CMAs 5 to 8 (minimum correlation coefficient 0.69) and between CMAs 5 and 6 (minimum correlation coefficient 0.86).

## Discussion

### Principal findings

In chronic conditions, where medications are taken over many years, EHRs and pharmacy records can serve as a proxy for adherence to treatment. 24.3% of person-years contained only a single prescription, resulting in several of the measures being non-calculable in these individuals. The supply duration was longer than the time between prescriptions for 49% of prescriptions, resulting in substantial variation between measures based on whether they used either the amount of medication acquired or the proportion of days with medication in supply. The correlation coefficients between the estimate of each adherence measure from one year to the next was highest for the CMA7 (0.58 – 0.66, depending on supply handling approach) and CMA8 (0.56 – 0.65) measures, indicating that in this population the estimates in a previous year would be appropriate as a proxy for current adherence in most people.

### Results in context

Even similarly defined measures for estimating adherence from EHRs may show poor agreement [50], highlighting their sensitivity to key underlying assumptions, and the importance of matching the adherence measure to its intended analytical purpose [51].

The measures described herein can be broadly categorised as measures of medication acquisition (CMA1 to CMA4) or medication availability (CMA5 to CMA8). Medication availability is more computationally complex than acquisition, and potentially sensitive to oversupply handling approach, however there is evidence to suggest that asthma controller medication availability may be more strongly associated with asthma clinical outcomes than acquisition [52], as it is the gaps in treatment which were particularly detrimental to asthma control.

These measures, CMA5 to CMA8, require specifying how over-supply should be handled. In this analysis, the average estimates at the year-level for each adherence measure were higher when over-supply was banked, and there was only a very small difference between the other approaches. We infer that in this population the cap was sufficient to manage outliers, without drastically affecting the findings for the average person.

There are also differences between the measures in how the end of the observation period is defined [53, 54]. Primarily, CMA1, 3, and 5 ended observation the day before the final prescription in the interval. As such, any long gaps after the last refill’s supply has been exhausted will be discounted. In EHR-based studies, we may have linked mortality records available, which facilitates identification of when someone died (and thus will not be needing further prescriptions). Additionally, changes in diagnosis or asthma resolution (common in childhood asthma [55] and occupational asthma [56]) may be recorded in primary care data. As such, we could confidently use measures which can be estimated with only a single prescription in the observation interval (e.g. not restrict ourselves to CMA1, CMA3 and CMA5).

### Limitations and future work

The primary value in estimating adherence from EHRs is in evaluating an individual’s *persistence*, including the duration and incidence of unscheduled treatment intermissions (an extended duration of consecutively missed doses, with the minimum duration varying by treatment and condition [57–61]). Intermissions may occur many times in the unbounded duration of asthma treatment, particularly as 30–50% of asthma patients in western Europe are classed as having intermittent asthma [62–64], according to the Global Initiative for Asthma (GINA) guidelines. Additionally, the most common reasons for a sanctioned treatment discontinuation are possible to identify in the EHRs, by searching for changes in prescriptions [29] or Read Codes relating to revised diagnosis, a change in regimen, or asthma resolution.

Using EHRs and pharmacy records serves as an indirect step to infer adherence: we cannot be certain that patients actually took the medication as prescribed, only that they collected their medication. However, particularly for chronic disorders with regular medication prescription needs, we argue this linkage of combination of EHR and pharmacy records can be considered a reasonably good proxy for adherence. Additionally, we are not able to detect *primary non-adherence*: cases in which a prescription was issued but never collected (as we only have dispensed prescription records) [65]. In England and Wales, however, the reverse would be true. As routine linkage to dispensing records is not possible in these countries (and others), it is not possible to detect which prescriptions were collected, and thus adherence estimation must be based purely on prescription records. In cases in which an individual repeatedly orders a prescription and never collects it, they may have an estimate of perfect adherence despite never having any medication ‘on hand’. Additionally, the developed methodology is currently only directly applicable to Scotland, because it has the existing infrastructure to enable linking prescribing and dispensing records, however the methodology can still be applied so long as the subsequent limitations are acknowledged.

Specifically relating to the methods employed herein, the primary limitation of this study is that the data linkage between prescribing and dispensing records in Scottish EHRs (conducted by National Services Scotland Information Services Division) is not a perfect process, as prescriptions containing multiple items have only a single identifier, rather than an item-specific identifier. As such, if the items are listed in a different order on the dispensing and prescribing records, additional information relating to a specific item (such as dosing direction notes from the pharmacist) may be assigned to the wrong prescription item. Although feedback and improvement to this system has resulted in improvement over time, the issue persists, and the incidence of such mismatching is hard to estimate. From a manual review of a sample of 1000 asthma medications included herein, less than 1% were obviously incorrect (either named a different medication or described a method of ingestion inherent to a different formulation, such as ‘inject’). Although rare, this mismatch is likely to have led to a small number of asthma-related records being erroneously excluded based on indication, as they contained exclusion keywords.

### Implications for policy, practice, and research

This paper describes various methods by which adherence can be measured in EHRs, providing insights to add nuance to the methodology selection for future works. This guidance will enable researchers to make more informed decisions about the methodology they employ, ensuring that adherence is captured in the most appropriate way for a certain study. This will facilitate stronger research evaluating the burden of non-adherence in specific populations, appraisals of new treatments and interventions, and prognostic models [66]. Ideally, this estimation may one day be integrated into both primary care and pharmacy systems to provide opportunity to encourage adherence at points of contact with patients [67]. Adherence measurement from prescribing data could also be employed by health insurance providers to incentivise better adherence, improving health outcomes while lowering costs for patients and the company.

Herein, we have used asthma corticosteroid adherence as a case study for an investigation into adherence monitoring using EHRs. However, there are several key learnings that can potentially be applied to other health conditions. Firstly, we have introduced new notation, as an extension to the work of Vollmer et al*.* [27], which specifies how medication supply should be estimated when there is theoretically supply remaining and a new prescription is filled. These approaches could also be extended to include the case of a change in treatment regimen. This will allow greater clarity in methods, and for comparing between different studies [25]*.*

Secondly, we have highlighted study design considerations which should affect adherence measure selection, such as whether it is possible to detect alternative reasons for treatment discontinuation (including surgical interventions, disease progression, and death).

Finally, this study provides detailed descriptions of simple rule-based data extraction from free-text entries, which can easily be modified for other health conditions. For example, extensions may be considered to include daily dose frequency relative to meal times (‘after food’) and alternative medication delivery mechanisms (‘inject’, ‘apply’, etc.).

## Conclusion

The measurement of medication adherence in EHRs can be conducted in many ways, with widely varied results. Careful consideration of the impact on risk of bias caused by the study design must be taken for each individual application and reported carefully. As far as we are aware, this is the first study to provide a systematic comparison of prescription record-based adherence measures. This study used asthma as a testbed for an investigation into adherence monitoring using EHRs, but our findings can in principle be adapted and may be generalisable across other chronic conditions.

## Supplementary Information

**Additional file 1: Appendix A.**

## Data Availability

The ALHS data are held by the National Services Scotland electronic Data Research and Innovation Service (eDRIS) in the National Safe Haven. Restrictions apply to the availability of these data, which were used under license for the current study, and so are not publicly available. Data would be made available from a reasonable request to phs.edris@phs.scot. Code scripts, in the R language, for all components of the data cleaning and analysis are available at github.com/hollytibble/EHR_Adherence.
