# Supplementary material for: Estimating medication adherence from Electronic Health Records: comparing methods for mining and processing asthma treatment prescriptions
Source: BMC Med Res Methodol. 2023 Jul 12;23:167. doi: 10.1186/s12874-023-01935-3 (PMC10337150; doi:10.1186/s12874-023-01935-3)
Supplement: Supplementary file 1 — Additional file 1: Appendix A. [file 12874_2023_1935_MOESM1_ESM.docx]

**Estimating Medication Adherence from Electronic Health Records: Comparing Methods for Mining and Processing Asthma Treatment Prescriptions**

**APPENDIX A**

**Asthma Medication Expected Supply Duration Calculations**

The expected supply duration of a prescription is a function of the amount that should be taken every day (the frequency of doses per day and the dose quantity) and the volume of the prescription.

**Dose Frequency**

**Example:** ‘TWICE A DAY’

The table below lists the keywords that were used to identify and classify patterns in the free-text dose directions.

First, any prescription records containing category A (‘Once’) keywords were identified. Of the remaining prescriptions, any with category B (‘Twice’) keywords or at least one keyword from both category C (‘Timing: Morning’) and D (‘Timing: Evening’) were identified as twice daily medications. Of the remaining, those with category E (‘Four Times’) were identified. Finally, of the remaining prescriptions, any containing category F (‘Daily’) keywords were identified. By doing these last we prevent ‘twice daily’ being identified as ‘daily’. Any remaining prescriptions which did not match any of these stated rules (from manual review, these were overwhelmingly those without recorded dose directions, although a minority were due to unclear or alternative regimens, such as six times daily) were imputed based on the most common values by drug: once daily for Ciclesonide and Fluticasone Vilanterol, else twice daily.

| **Daily Dose Frequency** | **Key Words** | | |
| --- | --- | --- | --- |
| 1. Once | "ONCE","O-D", "O.D" | | |
| 1. Twice | "TWICE", "TWO TIMES", "2 TIMES", "TD", "TID", "BID", "BD", "B-D", "B.D" | | |
|  | “MORN” | WITH | “NIGHT”, “EVE”, “BEDTIME” |
|  | “AM” |  | “PM” |
|  | “A.M” |  | “P.M” |
|  | “MANE” |  | “NOCTE” |
| 1. Timing: Morning | “MORN”, “MANE”, “AM”, “A.M” | | |
| 1. Timing: Evening | “NIGHT”, “EVE”, “BEDTIME”, “NOCTE”, “PM”, “P.M” | | |
| 1. Four Times | "QID", "FOUR TIMES", "4 TIMES" | | |
| 1. Daily | "DAILY", "EVERY DAY", "EACH DAY" | | |

**Dose Quantity**

**Example:** ‘TWO PUFFS’

The dose quantity was estimated by searching for the numbers one, two, three, or four (either in numerals or written out as above) preceded by “take” or “inhale”, or followed by “daily”, “at”, “to be taken”, or “puf” (with a single ‘f’ to allow for typographical errors), or “p” (followed by a space; ‘p’ being commonly used as shorthand for puffs).

The most common dose quantity by medication was also imputed when a value could not be extracted: one puff at each daily dose time for Budesonide, Ciclesonide, Fluticasone Vilanterol, Fluticasone Salmeterol, and Mometasone, else two puffs.

**Prescription Volume**

**Example:** 60 Puffs per cannister

In our dataset, the volume of doses prescribed was estimated by multiplying the number of units by the number of doses per unit (*unit volume*), extracted from the free-text prescription information. To do this, we searched for any of the values [200, 120, 112, 100, 60, 56, 50, 40, 30, 28, 24, 20, 14, 5] followed by any of “DOSE” (with or without a preceding space), “-DOSE”, or “ X ”. Additionally, records with quantity of 14 or over were included as extracted values of the prescribed doses. The next step was to impute values of unit volume for the records where information could not be extracted. Medications are frequently available in different pack sizes depending on the dosage, with lower strengths often available in larger volumes. As such, the modal value by strength, medication type and brand was imputed for missing values. If there were no records with extractable unit volume (and thus no mode could be calculated) the value was imputed as the smallest unit volume listed for that brand (or the most common brand for generic medications) and strength from the Electronic Medicines Compendium (EMC) website, medicine.org.uk, which hosts information on all medicines licensed for use the UK.

Each prescription record contained a field named ‘quantity dispensed’. In medications in tablet formulation, this is easily defined as the number of tablets dispensed. The interpretation for inhalers, however, could either be the number of doses in the cannister or the number of cannisters. Pragmatically, we require both, however we assumed that when the number of doses in the cannister was listed there was only a single cannister provided, or alternatively that the number recorded was equal the number of canisters multiplied by the number of doses in a cannister. As such, when the recorded quantity prescribed was less than 14, the medication dose quantity was calculated as the recorded quantity multiplied by the number of doses per pack, else it was simply the recorded quantity.

59.5% of prescriptions were for a single inhaler unit (cannister), 39.7% were for two units, and the remaining 0.8% were for 3 or more units. The doses per unit values were only identified for 15.2% of the records, and a manual review of the uncoded prescription records did not identify any further key phrases which would have improved the extraction process. The modal value (by medication, brand, and strength) could be imputed for an additional 83.6% of records (for a total of 98.8%). The values for the remaining 1.2% of prescription were imputed from the EMC (as described in the Methods) as follows: 120 doses for fluticasone formoterol or beclometasone formoterol inhalers, 100 doses for beclometasone inhalers, 60 doses for 500mcg fluticasone salmeterol inhalers, else 120 doses, and 60 doses for 400mcg budesonide formoterol inhalers, else 120 doses. The resulting estimated prescribed doses had a range of 14 to 2400, with a median of 200 (interquartile range 120 to 240 doses).
